# Utilization of Polymeric Materials toward Sustainable Biodiesel Industry: A Recent Review

**DOI:** 10.3390/polym14193950

**Published:** 2022-09-21

**Authors:** Fozy Binhweel, Mardiana Idayu Ahmad, Sheikh Ahmad Zaki

**Affiliations:** 1 Environmental Technology Division, School of Industrial Technology, Universiti Sains Malaysia, Penang 11800, Malaysia; 2 Renewable Biomass Transformation Cluster, School of Industrial Technology, Universiti Sains Malaysia, Penang 11800, Malaysia; 3 Malaysia-Japan International Institute of Technology, Universiti Teknologi Malaysia, Kuala Lumpur 54100, Malaysia

**Keywords:** polymers, biodiesel, polyhydroxyalkanoates, polymeric catalyst, CFIs, biodiesel exposure materials

## Abstract

The biodiesel industry is expanding rapidly in accordance with the high energy demand and environmental deterioration related to the combustion of fossil fuel. However, poor physicochemical properties and the malperformance of biodiesel fuel still concern the researchers. In this flow, polymers were introduced in biodiesel industry to overcome such drawbacks. This paper reviewed the current utilizations of polymers in biodiesel industry. Hence, four utilizing approaches were discussed, namely polymeric biodiesel, polymeric catalysts, cold-flow improvers (CFIs), and stabilized exposure materials. Hydroxyalkanoates methyl ester (HAME) and hydroxybutyrate methyl ester (HBME) are known as polymeric biodiesel sourced from carbon-enriched polymers with the help of microbial activity. Based on the literature, the highest HBME yield was 70.7% obtained at 10% H_2_SO_4_ ratio in methanol, 67 °C, and 50 h. With increasing time to 60 h, HAME highest yield was reported as 68%. In addition, polymers offer wide range of esterification/transesterification catalysts. Based on the source, this review classified polymeric catalysts as chemically, naturally, and waste derived polymeric catalysts. Those catalysts proved efficiency, non-toxicity, economic feasibility, and reusability till the 10th cycle for some polymeric composites. Besides catalysis, polymers proved efficiency to enhance the biodiesel flow-properties. The best effect reported in this review was an 11 °C reduction for the pour point (PP) of canola biodiesel at 1 wt% of ethylene/vinyl acetate copolymers and cold filter plugging point (CFPP) of B20 waste oil biodiesel at 0.08 wt% of EVA copolymer. Polymeric CFIs have the capability to modify biodiesel agglomeration and facilitate flowing. Lastly, polymers are utilized for storage tanks and auto parts products in direct contact with biodiesel. This approach is completely exclusive for polymers that showed stability toward biodiesel exposure, such as polyoxymethylene (POM) that showed insignificant change during static immersion test for 98 days at 55 °C. Indeed, the introduction of polymers has expanded in the biodiesel industry to promote green chemistry.

## 1. Introduction

The global production of biodiesel is escalating continuously since the end of the past century [1]. Based on the International Energy Agency (IEA), the annual biodiesel production was 53 billion liter in 2021. The expectation is to reach 56 billion liter in 2022. By the end of 2025, there will be a 30% anticipated increase in biodiesel production worldwide [2]. Such expanding production in biodiesel fuel is driven by the global giant desire to shift into more sustainable energy resources as a response to the high demand on energy sources in one hand, and to curb the deleterious environmental pollution on the other hand [3,4]. Biodiesel is a good alternative for conventional diesel since it has physicochemical properties close to fossil fuel-based diesel, making it a suitable substitute in diesel engines [5]. In addition to the suitability of biodiesel fuel to be poured directly in diesel engines without modifications, its lubricants characteristics resist the engine wearing out, which can be considered as a privilege for biodiesel compared with conventional diesel [6]. Furthermore, the higher content of oxygen in biodiesel leads to complete combustion of the fuel inside engine that results in lower unfavorable emissions [7]. Thus, biodiesel is classified as an ecofriendly alternative to the conventional diesel [8].

Biodiesel fuel is conventionally sourced from four generations of feedstocks. These feedstock generations include edible oils, inedible oils, algae oil, and genetically modified crop oils. Currently, more than 75% of the global biodiesel production is produced from the first-generation feedstock which requires wide cultivation areas [9,10]. In this case, first-generation biodiesel is not green enough since the total greenhouse gas footprint for both fuel combustion and cultivation emissions may exceed that of fossil fuel diesel [11]. Moreover, the accumulating costs of farming, cultivation, production, and treatment are not feasible economically since 70–80% of the biodiesel costs are accounted for by the feedstock expenses [12]. Beside the environmental impact and the economic feasibility, ethical issues concern researchers and decision makers. Large fertile areas, greater quantities of fertilizers, and significant water resources were dedicated to grow the feedstock of first-generation biodiesel. At the same time, the food shortage problem is encountered by many countries all around the world [13]. Therefore, expanding biodiesel production from first-generation feedstock is considered one of the main reasons behind food shrink and increasing food prices. The other three biodiesel feedstock generations seem more sustainable.

Despite being preferable environmentally and economically, second (inedible and wastes oils) and third (algae) generations of biodiesel feedstocks have drawbacks in characteristics and performance. The high acidity in the form of free fatty acids (FFA) is one of the distinguished characteristics of the second and third generation feedstocks lipids [14]. A higher content of FFA in the oils is an indicator for the rancidity of feedstock since it is almost waste. They come from oxidation reactions, moisture, effect of lipase enzyme, high temperature, and hydrolysis reactions within the feedstock itself [15]. FFA hinders the synthesizing fatty acid methyl esters during the transesterification reaction due to soap formation [16]. Another disadvantage of second-generation feedstock oil is its water content. The water exists naturally in the feedstock composition or finds its way through the purification process and absorption from the atmosphere. Water content in the oils promotes hydrolysis reaction which leads to a greater FFA content within the oil. Moreover, if the water content sneaks to biodiesel, it may be responsible for damaging some auto parts, particularly polymers due to attributing hydrolysis reaction, oxidation, and FFA formation [17]. Beside the existence of FFA and moisture, the higher content of saturated fatty acids (SFA) constitutes another barrier toward utilizing biodiesel fuel from second-generation, in particular animal-based feedstocks. The higher content of SFA leads to poor cold-flow properties in biodiesel fuel, which plugs the fuel in engine system and impacts negatively on the engine and its performance. Thus, a pretreatment process is needed for second-generation feedstock before subjecting it to biodiesel production.

Recently, polymers materials were introduced into biodiesel production to enhance the physicochemical properties and fuel performance, particularly for the second-generation feedstocks. Polymers are defined as series of molecules linked together by chemical bonds to form macromolecules. They exist naturally, e.g., proteins, wood, and rubber, and synthetically, e.g., nylon, polyethylene, and polyester [18]. They were introduced almost in every aspect of life including products of medicine, food, furniture, auto parts and so on. The biofuel industry was, and still is, one of the expanding sectors to introduce polymers materials to enhance the properties of fuel products. Polymeric biodiesel was synthesized biologically with the help of micro-organismic activity in presence of polymeric feedstock [19,20,21,22]. In addition, polymers were composited to reduce the higher content of FFA existed in second-generation feedstock oils. Moreover, they catalyze the esterification and transesterification reactions during production line of biodiesel fuel [9,10,23,24,25]. Beside catalysis, polymers were used as additives to improve the cold-flow properties and enhance the fuel performance [26,27,28,29,30]. Moreover, stable polymers were investigated as storage containers and auto parts in direct contact with biodiesel [17,31,32]. So, reviewing those studies would help to build a collective knowledge and solid background to valorize polymers in biodiesel production industry.

**Figure 1:**
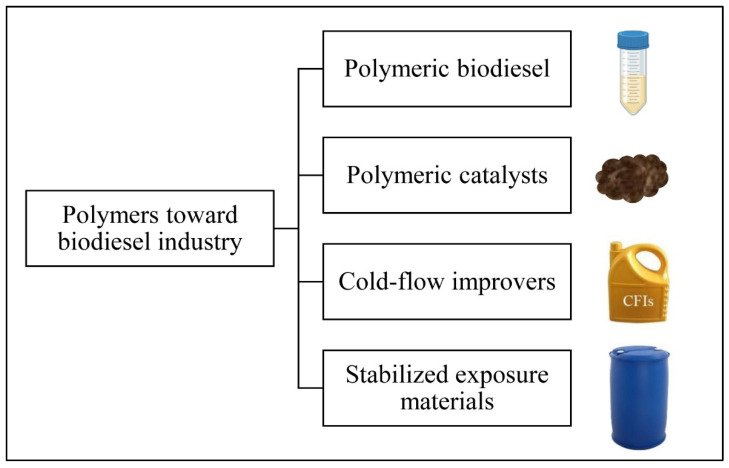
Utilization of polymers toward biodiesel industry.

Herein, recent studies regarding utilizing polymeric materials toward biodiesel industry were reviewed and depicted in Figure 1 as polymeric biodiesel, polymeric catalysts, cold-flow improvers, and stabilized exposure materials Biologically synthesized biodiesel from polymeric materials was explored. Production techniques, characteristics, and limitations were analyzed critically. Additionally, the polymeric catalysts were compared in terms of sources, catalytic efficiency, reusability, ecofriendly, and economic feasibility. Besides, different polymeric CFIs were discussed to improve the poor cold-flow properties of biodiesels particularly those rich in SFA. Discussion was developed about the stability of polymers used as storage containers and auto parts exposed to biodiesel fuel. The merit advantage of this work is to direct attention towards low-cost polymers as raw materials for biodiesel production, polymeric catalysts for several cycles of biodiesel conversion with efficient performance, effective polymers to enhance biodiesel physicochemical properties, and provide polymers featuring constant stability in direct contact with biodiesel fuel.

## 2. Polymeric Biodiesel

**Figure 2:**
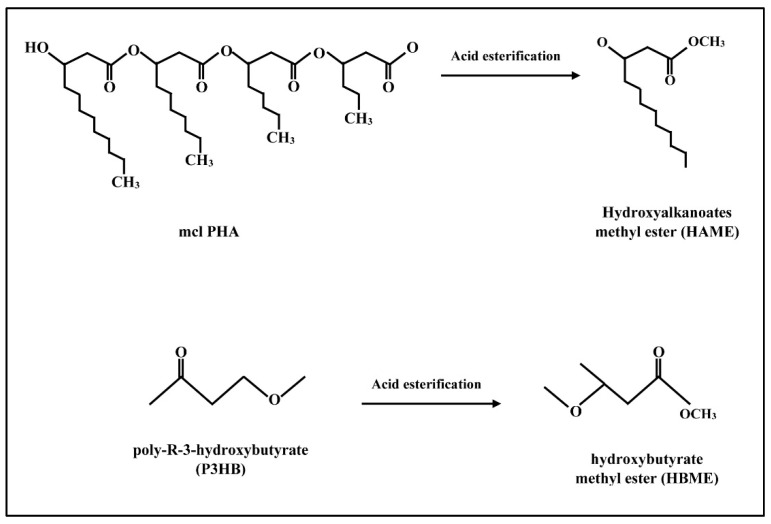
Production of polymeric biodiesel HAME and HBME from mcl PHA and P3HB.

Biodiesel fuel can be synthesized biologically based on polymers materials produced by particular species of microorganisms. These polymers are known as polyhydroxyalkanoates (PHAs). They are produced by prokaryotic microbes under particular nutritional conditions [33]. The bio-based polymers PHAs attracted the attention in multiple applications due to their preferable economic and ecofriendly characteristics which include biocompatibility, biodegradability, diversity, and sustainability. So, they have been used in bioplastic materials, medical applications, agricultural production, and theconstruction sector [34]. Recently, PHAs were applied to produce the biofuel. Biological-based biodiesel was first synthesized in 2009 by Zhang et al. [35]. The research group depended on PHAs polymers to synthesize hydroxybutyrate methyl ester (HBME) and hydroxyalkanoate methyl ester (HAME) through acid-catalyzed esterification for poly-R-3-hydroxybutyrate (P3HB) and medium chain length PHA (mcl PHA) as depicted in Figure 2. The recovery yields were 52% and 65% for HBME and HAME, respectively. Those HBME and HAME are close to the characteristics of biodiesel fuel. The calorific values were 20 MJ/kg and 30 MJ/kg for HBME and HAME, respectively. In the following discussion, the production techniques, characteristics, and limitations of polymeric synthesized biodiesel are itemized in detail.

### 2.1. Production Techniques

PHAs are polymeric ester-linked molecules consisting of considerable quantities of carbon and energy. Each molecule comprises 600–35,000 units of fatty acid monomers [36]. There are more than 150 recorded monomers, each monomer contains chain (R) group which are often saturated alkyl group or sometimes unsaturated or branched alkyl group. Based on the number of carbon atoms, PHAs are categorized into three groups, short (3–5 carbon atoms), medium (6–14 carbon atoms), and long (above 15 carbon atoms) chain length PHAs biopolymers. The difference in PHAs characteristics comes from the diversity of monomers. If the synthesis is made up of one type of monomer, it is called homopolymer while the synthesis of different types of monomers is called copolymer [19]. Table 1 shows examples of PHAs with the sources of carbon and synthesis microbes.

Under unbalanced nutrition conditions, prokaryotic microorganisms are responsible about synthesizing PHAs as stored intracellular matter [19]. There are more than 300 micro-organismic species, most of them are bacterial species, capable to produce PHAs. They were classified into two groups. The first bacterial group requires excessive carbon source and limited nutritional elements of nitrogen, sulfur, magnesium, and phosphorus. *A. eutrophus*, *Protomonas extorquens*, and *P. oleovorans* species are examples of this bacterial group. In contrast, the other group such as *Alcaligenes latus*, *Azotobacter vinelandii* and *E. coli* requires excessive sources of both carbon and the other elemental nutrients [53].

**Figure 3:**
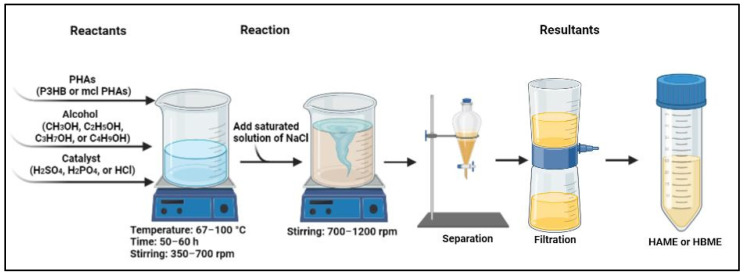
Schematic production of polymeric biodiesel HAME and HBME.

P3HB and mcl PHAs are the polymeric raw materials to synthesize the polymeric biodiesel HBME and HAME. They are sourced from rich carbonic source, such as dewatered activated sludge, and isolated using particular bacterial strains. The produced polymeric materials undergo esterification through acid-catalyzed hydrolysis, in which alcohol methanol or ethanol are added in optimized ratios to complete the conversion into alkyl esters. This chemical reaction is usually catalyzed by acid catalyst, and sulfuric acid (H_2_SO_4_) is used most frequently. However, hydrochloric acid (HCl) and phosphoric acid (H_3_PO_4_) were used to catalyze this chemical reaction. In addition to the acid catalysts, base catalysis was reported to synthesize HAME and HBME from mcl PHAs and P3HB polymeric materials. After the formation of HAME and HBME, saturated solution of salted water is added and stirred intensively. Then, the mixed solution is let to settle down into two layers. The more transparent layer is biodiesel HAME and HBME that should be dewatered and filtered to remove undesirable debris [19,35]. Since the raw materials PHAs for the polymeric biodiesel is non-toxic an utilized in medical, agricultural, and industrial applications, the wastes of this acid-catalyzed hydrolysis do not form threats [54]. However, a toxicity test is highly recommended for the polymeric biodiesel and byproduct wastes in future investigations. Figure 3 illustrates the production line of polymeric biodiesel (HAME and HBME).

There are four parameters affecting acid catalyzed esterification to synthesize polymeric biodiesel, i.e., the type and ratio of alcohol, type and ratio of the catalyst, reaction temperature, and reaction time. Four alcohols can be used in this chemical reaction, methanol, ethanol, propanol, and butanol. However, methanol and ethanol are the most frequently used due to their preferable physicochemical properties and low costs. It was reported that alcohol methanol is more efficient for converting mcl PHAs and P3HB into alkyl esters. The reason behind this efficiency is the short chain of the alkyl group within methanol which has higher solubility and activity. For alcohol ratio, higher amount of alcohol results in higher conversion percentage [19,55]. For the catalyst type and ratio, H_2_SO_4_, H_3_PO_4_, and HCl acids were used to catalyze the esterification reaction. Literature reported usage of base catalysts for this reaction as well. Among all catalysts, H_2_SO_4_ was the most frequently used to yield high percentage of HAME and HBME. The literature reported that the maximum yield of polymeric biodiesel can by synthesized with a catalyst ratio 10–15% [19,50,56]. For the temperature parameter, it is normal to adjust the reaction temperature around the boiling point of alcohol in order to avoid alcohol evaporation. Choonut et al. [21], Keunun et al. [22], and Sangkharak et al. [20] obtained the highest yield of HBME at 67 °C. However, Zhang et al. [35] got the maximum HBME at 100 °C. For time parameter, much time is required for this esterification process. According to Choonut et al. [21], the highest HBME percentage was produced from P3HB at a reaction time 50 h. Further increasing time led to a decrease of HBME yield. Table 2 explores the previous studies regarding HAME and HBME production with optimum values.

### 2.2. Characteristics and Limitations

Polymeric biodiesel (HAME and HBME) has physicochemical properties almost close to biodiesel fuel. In spite of being sourced from low-cost feedstocks like sewage sludge or wastes, HAME and HBME offer valuable, ecofriendly, and sustainable biodiesel fuel. Unlike vegetative and animal-based biodiesel, the polymeric biodiesel does not require extensive purification process. The calorific value of this pure biologically derived fuel reached 20 and 30 MJ/kg for HBME and HAME, respectively. The heating value can be improved to 30 and 35 MJ/kg for HBME and HAME respectively by adding 10% ethanol [35]. The polymeric biodiesel has preferable performance characteristics. The start ignition is faster than vegetative and animal-based biodiesel. In addition, just short combustion time is needed to fully burn. Plus, this fuel produces very little trace amounts of exhaust smoke. The reason behind these preferable performance characteristics refers to the high oxygen content of HAME and HBME fuels which helps to combust the full fuel within short time and produce neglectable smoke. Besides, the polymeric biodiesel contains neither nitrogen nor sulfur. That is why its emissions are more ecofriendly since it is free of nitrogen oxides and sulfur oxides. Thus, sustainable biodiesel fuel with favorable characteristics is offered by biopolymers materials derived from low-cost feedstocks which are not competitive with food resources [19]. Table 3 explores the characteristics of polymeric biodiesel HBME.

Despite the preferable characteristics of biologically derived polymeric biodiesel, there are some drawbacks that may limit using HAME and HBME as an alternative fuel. The conversion percentage of mcl PHAs and P3HB into alkyl esters is lower compared with vegetative and animal-based biodiesel. Zhang et al. [35] recorded conversion percentages for the first time as 52% and 65% for HBME and HAME, respectively. However, 70% recovery yield was recorded later by Choonut et al. [21], Keunun et al. [22], and Sangkharak et al. [20]. Beside the low conversion percentage, much time is required to reach the maximum yield. The optimized results of Choonut et al. [21] revealed that only 12.8% yield of HBME can be obtained from P3HB after 10 h of esterification reaction. The yield increased to 70.7% once the reaction time was doubled five times into 50 h. However, the HBME yield was decreased beyond 50 h of reaction time. The polymeric synthesized esters of HBME and HAME have a lower cetane number compared with conventional diesel and biodiesel since they are highly oxygenated compounds. Moreover, their vaporization latent heat is high. Thus, much enhancement is needed before fueling engines with polymeric biodiesel [35].

## 3. Polymeric Catalysts

**Figure 4:**
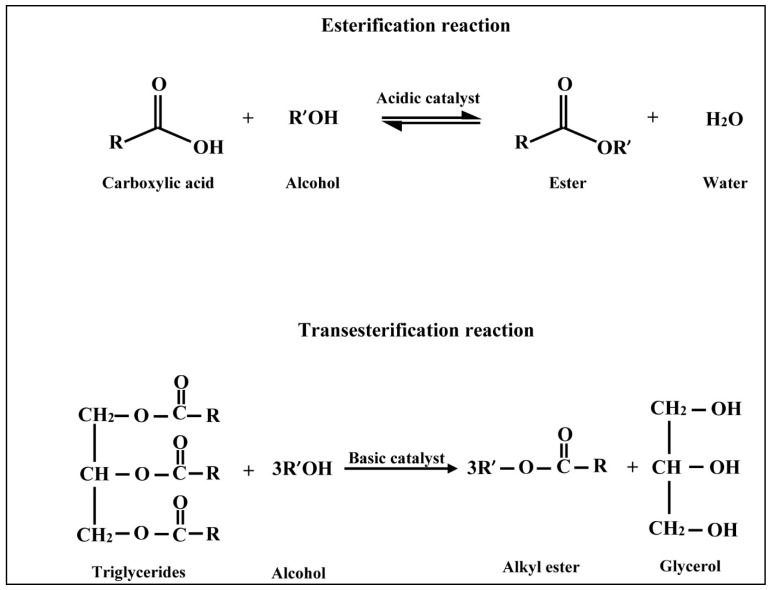
Esterification/Transesterification reactions to produce biodiesel fuel.

Limitless catalysts were reported in the literature to catalyze the conversion process of triglycerides into alkyl esters. The conversion is done through esterification and/or transesterification chemical reactions illustrated in Figure 4 [58]. The mechanism of both reactions has similarities and differences. In acidic esterification, carbonyl group of triglycerides reacts with the catalyst to convert triglycerides into diglycerides in the first reaction. Diglycerides are converted into monoglycerides in the second reaction, and esters in the third reaction. In contrast, the catalyst reacts with alcohol in basic transesterification to form alkoxy group. Then, the three reactions take place between alkoxy group and triglycerides to be converted into diglycerides, monoglycerides, and finally the alkyl esters [9,59]. Catalyst functions to speed these processes 4000 times [60]. In general, catalysts were classified into homogenous and heterogenous catalysts. Homogenous catalysts are chemical compounds that have the solubility characteristic in either lipids or alcohol during biodiesel production. They offered high efficiency in mass transfer of the catalytic group. That is why the homogenous-catalyzed transesterification does not consume much time. However, contamination of the final products constitutes a drawback for homogeneous catalyst. Consequently, a purification process is highly needed to separate catalyst from resultants. However, the reusability of homogeneous catalysts is not promising. In contrast, heterogeneous catalysts are insoluble compounds in lipids nor alcohol. Therefore, separating heterogeneous catalyst from resultants is not challengeable which saves resources and time in the purification process. In addition, the reusability property is potentially attainable. Compared with homogenous catalysts, heterogenous catalysts consume much time to catalyze the conversion process of triglycerides into biodiesel since they offered poor mass transfer characteristics of the catalytic group [61,62].

Polymers materials offered efficient catalysts for biodiesel production. Indeed, the polymeric catalysis is rapidly expanding in accordance with the revolution of green chemistry. The performance efficiency of polymeric based catalysts comes from the interior porous structure of polymers that provides a wide surface area and diffusible capability, selectivity functions of polymers materials, high stability of polymers compounds, and the unique chemical composition of polymers which allows for some adjustments [24]. Polymeric catalysts are produced through chemical, natural, and waste derived composites. All these composited approaches proved high efficiency in catalyzing triglycerides transesterification and FFA esterification, particularly for the second-generation feedstock oils. They provide high mass transfer of the catalytic group during reaction which allows for high conversion yield of fatty acids methyl esters (FAME) within less reaction time. Moreover, polymers, both homogeneous and heterogeneous, migrate completely to glycerol byproduct. In this case, purification process of produced biodiesel is unrequired. In addition, the recovered catalyst is reusable for several cycles with high conversion percentages as reported in the literature [10,23]. In this review, polymeric catalysts are classified into three groups, i.e., chemically synthesized polymeric catalysts, naturally derived polymeric catalysts, and waste derived polymeric catalysts.

### 3.1. Chemically Synthesized Polymeric Catalysts

Chemosynthetic polymeric catalysts are commercialized polymeric chemicals synthesized in the lab based on specific parameters and conditions to catalyze triglycerides transesterification and FFA esterification. There is a wide range of chemosynthetic polymeric catalysts that include acidic, basic, organic, inorganic, and hybrids of organic-inorganic, porous, and metals. FDU-15-SO_3_H is an example of acidic organic pours polymeric catalysts. H_2_SO_4_ was the agent to sulfonate FDU-15 polymer. This polymeric composite with large surface area 447 m^2^/g proves efficiency to convert 99% of soybean oil into FAME in the first run and 90% in the fourth cycle [63]. Polymeric ionic liquids were introduced as an ecofriendly catalysis approach in a variety of industrial chemistry including biofuel production. Poly divinylbenzene (PDVB-IL) is an ionic liquid catalyst synthesized through copolymerizing acidic ILs oligomers and DVB. The conversion efficiency for PDVB-IL was 99% and the reusability efficiency was 89% in the sixth cycle [64]. Moving to basic catalysts, resin-N_3_=P(MeNCH_2_-CH_2_)_3_N was synthesized by Reddy et al. [65]. The more interesting thing about this polymeric basic catalyst is the highest efficiency, which reached 100% to convert soybean oil into biodiesel within 3 h at room temperature, 25 °C. Another unique polymeric catalyst is poly (butanesulfonic acid pyrrole) coated magnetic iron oxide. It was synthesized by a hybrid mixture of organic/inorganic chemicals. The most important feature of this hybrid polymeric catalyst is the rate of reusability which reached 97.5% in the tenth cycle [66]. Metal-organic frameworks were modified to synthesize MIL-100(Fe)@DAILs catalyst. It was synthesized by encapsulating dicationic acid into MIL-100(Fe) framework [67]. The redox property of polyoxometalates was utilized to synthesize acidic heterogeneous catalyst HPW@MIL-100(Fe) which proves 96.3% efficiency to esterify FFA into FAME with reusability capability 95.5% in the fifth cycle [68]. Moreover, the alkalinity property of some metal-organic frameworks was utilized to prepare an alkaline polymeric acid Fe_3_O_4_@HKUST-1-ABILs that can trans-esterify 92.3% of soybean oil into biodiesel fuel [69]. 90% of oleic acid can be esterified by HZnPS-1 catalyst which was synthesized by optimizing the chemical reaction of p-xylenediphosphonic acid and anhydrous ZnCl_2_ and sulfonating the obtained resultants [70]. Oligocat was the given name for the novel catalyst synthesized by Vlnieska et al. [9,10]. It behaves as a pseudo-homogeneous acidic catalyst, demonstrating 96.5% conversion efficiency even after the third cycle. Lastly, Pd/HPS catalytic polymeric material was prepared by immobilizing palladium nanoparticles into hyper-crosslinked polystyrene. These kinds of polymer-metal catalysts were used to catalyze the hydrodeoxygenation reaction, which is a new approach to produce hydrocarbon-based biodiesel. Sapunov et al. [71] used Pd/HPS to catalyze the hydrodeoxygenation reaction of stearic acid which led to 97% conversion into n-heptadecane. Table 4 explores the conversion and reusability efficiencies of some chemically synthesized polymeric catalysts.

### 3.2. Naturally Derived Polymeric Catalysts

Naturally derived polymeric catalyst are natural polymers with a little modification that have the ability to catalyze the triglycerides transesterification and FFA esterification reactions. Those kinds of polymeric materials are considered as green, recyclable, and biodegradable catalysts. Despite being supportive for the green chemistry, the naturally derived polymeric catalysts are feasible economically since they are derived from low-cost feedstocks. Chitosan is one of the most abundant natural polymers. It can be extracted from marine crustaceans, insects, and fungi. Natural chitosan can be used as a catalyst for biodiesel production. The functionality of chitosan comes from hydroxyl and amino groups obtained from alkaline deacetylation of amide groups of chitin (poly(β-(1-4)-N-acetyl-D-glucosamine) [24,72]. da Silva et al. [73] adsorbed Cu ions into chitosan to synthesize CCu catalyst which was employed on babassu oil under optimized reaction conditions to yield 76.8% biodiesel. However, the yielded biodiesel percentage was increased to 97% when chitosan immobilized with CaO and employed on soybean oil [74]. The increased thermal and mechanical stability of chitosan with supportive CaO may be the reason behind increasing biodiesel production once this basic polymeric catalyst was used in the transesterification reaction [24]. In contrast, other heterogenous acidic catalysts were successfully synthesized from natural chitosan. Sulfonic group -SO_3_H was inserted into chitosan structure once the sulfosuccinic acid (SSA) was crosslinked into chitosan. The new polymeric composite successfully yielded 89% of palmitic acid under optimized parameters [75]. HTCC/Na_2_SiO_3_/ECH catalyst was synthesized by interlinking sodium silicate Na_2_SiO_3_ and chitosan chloride (HTCC) with epichlorohydrin (ECH). The nanoparticles composite of Na_2_SiO_3_ formed bridge to chitosan which led to increase the catalytic activity and biodiesel yield to 97% from soybean oil [76]. Beside chitosan, alginate is another naturally occurring biopolymer derived from brown seaweeds and marine algae [77]. With a little modification, alginate have been used to catalyze esterification/transesterification reactions toward biodiesel production. Boey et al. [78] synthesized ferric alginate to esterify lauric acid. They obtained 99% conversion percentage of methyl laurate. More interestingly, Naz et al. [79] prepared Tin (Sn^2+^) alginate catalyst to esterify oleic acid with conversion efficiency 98.7 and reusability possibility till the eighth cycle with 97.6% efficiency. Cellulose is another naturally occurring polymeric material sourced from plants wood that can be used in biodiesel catalysis. In addition to the availability and feasibility, other characteristics, e.g., biocompatibility, modification suitability, and porosity, make cellulosic materials high featured catalysts [24]. CB-(AST-HPW) catalyst was synthesized from porous cellulose beads. The catalyst successfully esterified 96% of yellow horn oil under optimized conditions with 70% efficiency after seventh cycle [80]. Cellulosic nanocomposite was modified and subjected to magnetic adsorption of hybrid organic/inorganic acids to produce sulfonated cellulose-magnetite nanocomposite (MSNC) catalyst. The cellulosic nano-catalyst had an efficiency to esterify 96% of oleic acid in the first run and 95% in the fifth cycle [25]. Enzymes are also considered naturally derived catalysts. Lipase enzyme is widely used as a catalyst for biodiesel production. It is usually extracted from the activity of living things. Recently, lipase was immobilized onto polymeric and inorganic materials to increase the efficiency and decrease the costs of lipase extraction [24]. Lipase PS was extracted from species *Burkholderia cepacia* and capsulated inside κ-carrageenan biopolymer. Then, it was used as a catalyst for the palm oil transesterification reaction. The obtained result is a complete conversion of palm oil into FAME. The polymeric enzymic catalyst revealed 82% efficiency after the fifth cycle [81]. Another lipase enzyme extracted from *Aspergillus niger* was supported on UiO-66 MOF and coated with polydimethylsiloxane (PDMS). The synthesized polymeric catalyst, ANL/UiO-66-PDMS-6 h, has conversion efficiency of 88% in the first cycle and 83% in the tenth cycle [82]. Table 5 explores the conversion and reusability efficiencies for some naturally synthesized polymeric catalysts.

### 3.3. Waste Derived Polymeric Catalysts

They are polymeric wastes utilized and modified to synthesize useful forms of polymeric materials with the purpose of catalyzing triglycerides transesterification and FFA esterification. Caetano et al. [83] synthesized D50w2 catalyst from polystyrene cross-linked divinylbenzene and sulfonated with -SO_3_H. It was employed on palmitic acid to yield 76.8% biodiesel under optimized conditions. Similarly, oleic acid was esterified by the acidic polymeric catalyst D5081 and yielded 97% biodiesel. This acidic polymeric catalyst was utilized from waste hyper-cross-linked polystyrene and improved with sulfonic group. The reusability efficiency reached 92% after the fourth cycle [84]. Low conversion efficiency was observed with partially sulfonated polystyrene polymeric acidic catalyst PSS. Only 53.4% of the oleic acid was barely converted to biodiesel in the first run. Additionally, the efficiency was reduced till 35.1% in the third cycle [85]. In contrast, the conversion efficiency reached 100% with polystyrene-supported fluoroalkyl superacid sPS-S. Moreover, the reusability efficiency remains considerable till the tenth cycle where it reached 88% once it was employed on Dodecanoic acid [86]. MSA-Pani catalyst was synthesized from methanosulfonic, modified polyaniline, and organo-sulfonic acidic groups. The acidic composite revealed efficiency 92% to covert Ricinoleic acid into biodiesel [87]. p-toluenesulfonic acid-functionalized polyaniline was utilized to synthesize the acidic polymeric catalyst *p*-TSA–PANI. This catalyst proves good efficiency and reusability possibility where it employed on waste cooking oil and yield biodiesel 97.1% in the first run and 94.3% at the tenth cycle [88]. Recently, waste scraped tires were utilized to synthesize heterogeneous polymeric solid acidic catalyst TPC-SO_3_H. This catalyst was prepared by carbonization of waste polymer tires. Then, it was sulfonated with strong H_2_SO_4_ to obtain the novel catalyst. TPC-SO_3_H was employed on chicken fats which featured with high content of FFA. Even though, the heterogenous polymeric catalyst proves high conversion efficiency 98.8% in the first run and 92.5% in the third cycle. However, the conversion efficiency declines till 48% in the seventh cycle [23]. Table 6 explores the conversion and reusability efficiencies for some waste derived polymeric catalysts.

**Figure 5:**
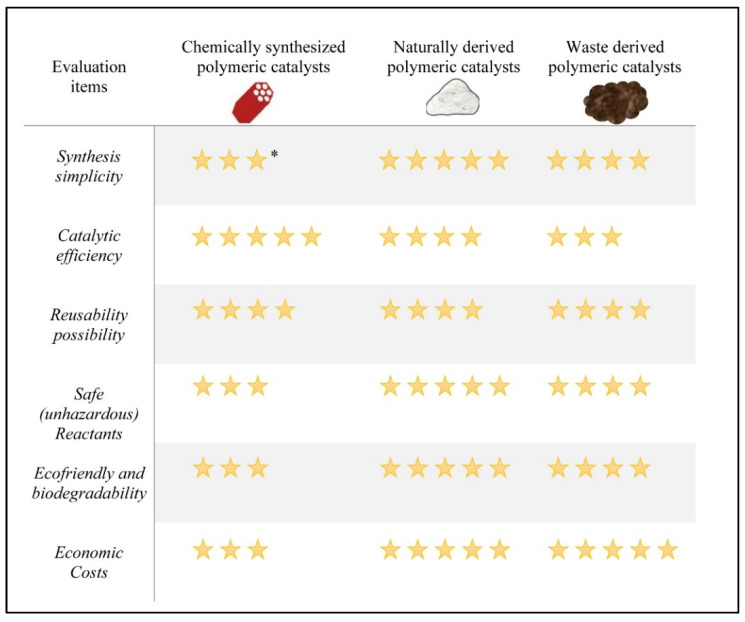
Overall evaluation of biodiesel polymeric catalysts. * Stars refer to the goodness of the evaluated characteristic, 5 stars mean very good, 4 stars are good, and 3 stars refer to acceptable evaluation.

Evaluation of the three groups of polymeric catalysts for biodiesel production have been carried out as depicted in Figure 5. The evaluation was conducted based on six items, synthesis simplicity, catalytic activity, reusability possibility, safe reactants, ecofriendly and biodegradability, and economic costs. Chemically synthesized polymeric catalysts have excellent catalytic activity. However, other aspects remain concerning since they contain toxic reactants that may constitute a medium hazard for the personnel and environment. Moreover, the complexity of chemical synthesis makes the chemosynthetic polymeric catalysts unpreferable. In contrast, the naturally derived polymeric catalysts feature simplicity in preparation, biodegradability, and affordable economic costs. Similarly, the waste derived polymeric catalysts are low-cost materials utilized for biodiesel catalysis. Beside the catalytic characteristics, the superiority of the last approach is to reduce the accumulated polymeric wastes from the environment.

Reusability is one of the main advantages of the three groups of chemically, naturally, and waste derived polymeric catalyst in biodiesel industry. Most of the reported polymeric catalysts proved reusable efficiency for several cycles. However, the performance of reused catalyst declines compared with the first run. The catalysis reactivity depends on the polymer chemical composition and the cycles number [89]. Synthesized polymers, e.g., Poly (butanesulfonic acid pyrrole) coated magnetic iron oxide, ANL/UiO-66-PDMS-6 h, sPS-S, and p-TSA–PANI have reusable activity reached 10 cycles with remarkable yield 97.5%, 83%, 88%, and 94.3, respectively [66,82,86,88]. This reusability characteristic of polymeric catalysts promotes the circular economy to produce sustainable biodiesel with low costs.

## 4. Cold-Flow Improvers

The flow behavior of oils is known as cold-flow properties. This scientific term includes three properties which are cloud point (CP), pour point (PP), and cold filter plugging point (CFPP). Cloud point is the lowest temperature at which small crystal shapes with hazy color start formation. Regarding pour point, it is the lowest temperature at which the liquid oil resists pouring and stops tilting. Lastly, the cold filter plugging point is defined as the ability of specific oil volume to pass through particular wired mesh during given time. All the three characteristics of cold-flow properties are usually measured based on standardized methods of the American Society for Testing and Materials (ASTM), e.g., ASTM D2500, ASTM D97, and ASTM D6371 for CP, PP, and CFPP, respectively [26,90].

Cold-flow properties of biodiesel are greatly influenced by fatty acids composition and existence of micro-impurities. The basic units of biodiesel fuel are the fatty acids alky esters. Those fatty acids were classified based on saturation situation into saturated fatty acids (SFA) and unsaturated fatty acids (UFA) which includes monounsaturated fatty acids (MUFA) and polyunsaturated fatty acids (PUFA). UFA are usually sourced from vegetative first-generation feedstocks whereas the SFA are obtained from second and third generation feedstocks. Melting point is one of the main features that can distinguish between the two types of fatty acids. SFA usually have melting points between 4.3–58.6 °C which are much higher than the UFA melting points that range between −1 to −52 °C. Accordingly, the components and ratio of fatty acids are the main reason behind the cold-flow properties. The higher the SFA contained in biodiesel fuel, the higher cold-flow properties [91,92]. Beside the fatty acids’ composition, micro-impurities influence cod-flow properties as well. It was reported that some biodiesels contain monoacylglycerols and steryl glucosides compounds. These two compounds have high melting points, reaching 70 °C for monoacylglycerols and exceeding 240 °C for steryl glucosides. So, they are solidified fast and form crystals shapes in liquified biodiesel fuel [93,94].

Poor cold-flow properties impact negatively on diesel engine systems, particularly in cold weather regions. Start-up problems are usually related to the cold-flow properties. Such problems arise when the diesel engine is turned off over night or stops working for long time. As a result of cold weather, biodiesel fuel solidifies in pipes and filters. Consequently, the fueling system will be plugged. Besides, poor cold-flow properties of biodiesel lead to poor performance of the diesel engine since the fuel supplies are hindered. In addition, engine damage is another concern related to the poor CP of biodiesel. The crystal particles formed during lower temperatures may make the fuel lose its flowability and engine loses its operability. Based on that, treating the poor cold-flow properties is an essential procedure for biodiesel in particular and fuel in general to come up with standardized physicochemical properties and well-operated performance [95,96].

There are three practical approaches to enhance the poor cold-flow properties of biodiesel, i.e., blending with conventional diesel fuel or other similar fuels having good cold-flow properties, winterization, and using additives. Literature reports acceptable cold-flow properties for biodiesel fuel when it was blended with fossil fuel diesel, kerosene, ethanol, and other good cold-flow biodiesels [95,97]. Winterization is a thermochemical fractionation process used to remove undesirable parts of oils. It is conducted based on the differences in melting points where the fuel is subjected to specific low temperature points till crystallized shapes are formed. Then, the solidified parts, which are responsible for the poor cold-flow properties, are filtered and avoided [98]. The last approach is using additives to enhance the biodiesel’s cold-flow properties. Multiple kinds of additives have been reported in the literature such as metal additives, oxygenated-agents additives, antioxidants additives, and CFIs. Literature reported enhanced performance and economic benefits as a result of using additives to enhance cold-flow properties of biodiesel fuel [99,100]. In this regard, polymers were used in biodiesel industry as CFIs. Polymers additives create modification in the structural agglomeration of biodiesel crystals at lower temperatures. With the polymeric materials, crystals take the needle shape, which eases the flowability through filters, pipes, and injectors in the engine’s fueling system [101]. Here, there are two types of polymeric CFIs, the first type is for the pure biodiesel and the other is used for blended biodiesel/diesel fuel.

### 4.1. Polymeric CFIs for Pure Biodiesel

Different polymeric materials have been used as CFIs for biodiesel. Wang et al. [102] used commercial polyolefins CFI to enhance cold-flow properties of biodiesel synthesized from waste cooking oil. The findings proved the capability of 0.04 wt% T803 polyolefins to reduce CP and CFPP 1 °C. Similarly, Nie and Cao [27] used multiple industrial polyolefins in a unified concentration 0.1 wt% on waste cooking oil biodiesel. The reduction was 2–3 °C in CFPP depending on the industrial polyolefins type. Beside polyolefins, ethylene/vinyl acetate (EVA) copolymers proved better progress as CFIs for biodiesel fuel. J. Wang et al. [102] could reduce CFPP and PP of waste cooking oil biodiesel by 2 and 6 °C, respectively, once they used 0.02–0.08 wt% of EVA. The same concentration of EVA with same biodiesel feedstock was used by Cao et al. [103]. However, PP was lowered 3 °C. In addition, EVA was used as CFIs for soybean biodiesel in a concentration of 0.01 wt%. The PP was reduced 2 °C [104]. The remarkable PP reduction was achieved by Chastek [105] once EVA was used with a concentration of 1 wt% on canola biodiesel to reduce PP 11 °C. In addition, blended biodiesel of rapeseed and soybean oils were subjected to 0.3 wt% Keroflux (BASF), EVA as CFIs. Here, 2 and 9 °C were the reduced values of CP and CFPP, respectively [28]. Two types of polyacrylates and related, copolymers poly(dodecyl methacrylate) and poly(hexadecyl methacrylate), have been tested as CFIs for canola biodiesel with a concentration of 1 wt%. Both of them recorded successful reduction in PP to −46 and −20 °C, respectively [105]. Moreover, 0.02–0.08 wt% of polymethyl acrylate have been tested on waste cooking oil biodiesel by Wang et al. [102] which lowered 8 °C in PP. The last group is maleic anhydride copolymers and their derivatives, from which two types poly(MA-alt-1-octadecene) and octadecyl vinyl ether were tested. The first one was tested on palm oil biodiesel [106] and tobacco seed biodiesel [107]. The results were a reduction of 6 °C in PP for palm oil biodiesel and 7 °C in CFPP for tobacco seeds biodiesel with concentrations of 2 and 1 wt%, respectively. The other one succeeded to reduce 3 °C in PP once it was tested on canola biodiesel with a concentration of 1 wt% [105]. Table 7 explores more details regarding polymeric CFIs for pure biodiesel.

### 4.2. Polymeric CFIs for Blended Biodiesel/Diesel

Despite the enhancement achieved by blending biodiesel with diesel fuel, using polymeric CFIs recorded better improvement. Xue et al. [30] applied copolymers of C9–C22 α-olefins on a blend of waste cooking oil biodiesel and diesel. 0.04 wt% of referred polymeric CFIs reduced 8 °C in CP and 7 °C in PP. Similarly, B20 of waste cooking oil biodiesel was subjected to 0.08 wt% of EVA copolymer. The results recorded reduction 8, 10, and 11 °C in CP, PP, and CFPP, respectively [103]. EVA copolymer was also applied on B50 of rapeseed biodiesel with 320 ppm as a concentration. However, the enhancement was remarkable in CFPP, at 17 °C [108]. B20 blending of coconut biodiesel gained improvement in cold-flow properties, specifically 3, 8, and 9 °C in CP, CFPP, and PP, respectively when 0.03 wt% of poly(methyl acrylate) was added to the blended fuel [109]. Lastly, poly(acrylic acid) and poly(tetradecyl methacrylate) were added to a B20 blend of palm oil biodiesel with a concentration of 0.1 wt%. The result recorded a 7 °C reduction in PP [29]. Table 8 summarizes the literature regarding the application of polymeric CFIs for blended biodiesel/diesel fuel.

## 5. Stabilized Exposure Materials

Pure or blended biodiesel, as a liquid fuel, needs to be contained within tanks during production, transportation, storage, and application stages. In this case, the tanks and containers materials would be exposed to biodiesel fuel for long time. However, there is a concern about the materials in direct contact with biodiesel fuel whether they are storage tanks or auto parts in the fueling system. The acidity and FFA content in biodiesel fuel may deteriorate the containers or auto part materials over time. These deteriorating factors are either inherent from the raw feedstock or formed during hydrolysis reactions in the fuel body. With the help of heat, light, metals, and peroxides, unsaturated alkyl esters can be oxidized and formed organic acids and other deteriorating factors in biodiesel fuel. Beside wearing out tanks and auto part materials, the existence of organic acids and FFA diminishes biodiesel physicochemical properties and leads to poor performance of the fuel [17,110,111].

Polymers are widely used in auto parts and storage tanks because of desirable properties of durability, affordability, and stability-flexibility nature. However, some polymeric materials revealed a negative effect once they were exposed to biodiesel fuel. The incompatible contact between biodiesel and polymers results in changes in physical characteristics like swelling, chemical characteristics like the structural composition, and mechanical characteristics like hardness and softness. The physical and chemical deterioration is driven by the polymers’ affinity to absorb and react with biodiesel solvent. This behavior maximizes the mass and volume of the polymeric material which is known as swelling. Consequently, the chemical structure of polymers would be affected, leading to another change in the mechanical nature of polymeric material represented in hardness, stiffness, thickness, elongation, and tensile stress [31,112].

Static immersion test is conducted to a variety of polymers in order to test the stability toward biodiesel exposure. The results revealed different stability responses depending on the polymeric material itself. In this regard, polymers can be classified into two groups, stable and unstable polymers, towards biodiesel exposure as it is explored in Table 9 and Table 10. Maru et al. [113] conducted the static immersion test for high-density polyethylene (HDPE) on soybean and sunflower biodiesel at 60 °C. The polymeric HDPE gained 5% of its initial weight after 125 days, likewise the results of Baena et al. [17] where the HDPE gained 5% after 98 days at 55 °C when it was immersed in palm biodiesel. The same test procedures were implemented on polyamide 66 (PA66). However, the polymeric materials showed 2% reduction from the initial weight. Polyethylene (PE) and polyoxymethylene (POM) showed weight reduction as well. Around 1% of their initial weight was lost after 28 days of immersing in a mixture of biodiesel from different feedstock sources at room temperature [114]. In addition, polytetrafluoroethylene (PTFE) lost weight after 41 days of immersing in blended palm biodiesel/diesel at room temperature as well. However, hardness and tensile stress of ethylenepropylenediene monomer (EPDM) have been weakened once they subjected to almost the same test parameters [32]. The instability was recorded for nylon 6/6, nitrile rubber, and high-density polypropylene within 29 days of immersing in blended biodiesel at 51.7 °C [115]. Table 9 explores the polymeric materials that showed instability toward biodiesel exposure.

In spite of instable polymers abundance, there are some polymers that showed stability toward biodiesel exposure. Teflon, Viton A401-C, and Viton GFLT were subjected to static immersion test on blended biodiesel. There was no recorded change in physical properties after 29 days of immersion at 51.7 °C [115]. Moreover, Haseeb et al. [31] immersed nitrile rubber, polychloroprene, and fluoro-Viton A in palm biodiesel for around 20 days at 50 °C. However, there were no significant changes in the polymeric materials. Likewise, POM recorded no significant change after immersing in palm biodiesel at 55 °C for 98 days [17]. Table 10 explores the polymeric materials that showed stability toward biodiesel exposure.

## 6. Economic Feasibility

Economic development is strongly connected with energy availability. Affordable energy resources make all the difference regarding sustainable economic growth [116]. Hence, biodiesel fuel contributes significantly as an alternative fuel for petroleum-based diesel since 53 billion liters of renewable biodiesel were produced in 2021 [2]. Considering 70–80% costs of biodiesel production come from the raw materials, low-cost raw materials would be more feasible economically [12]. In this respect, polymers represent a low-cost feedstock to synthesize polymeric biodiesel fuel. Those polymeric raw materials are sourced from carbonic-rich wastes and organic matters. Beside affordability, polymeric raw materials feature biocompatibility, biodegradability, and sustainability.

The economic analysis of biodiesel production includes the catalyst costs. In order to reduce the costs of final biodiesel product, low-cost catalysts are an essential factor toward sustainable and affordable biodiesel industry. Polymers, in particular naturally and waste derived, offered low-cost composites to catalyze FFA esterification and triglycerides transesterification reactions. Indeed, such an approach promotes green chemistry since it reduces the accumulated wastes and utilizes the natural materials in energy industry. Beside the catalytic materials, the reusability characteristic of polymeric catalysts enhances the reduction of biodiesel production costs. Polymer-derived catalysts proved efficiency for converting triglycerides into alkyl esters for several cycles which helps to save the resources and reduce the production costs [24,89].

Avoiding the negative impact of the final product is accountable in the economic evaluation [116]. In this regard, polymers offered composites to enhance the physicochemical properties of biodiesel that would boost the fuel performance and curb the combustion emissions. In addition, polymers materials that showed stability in exposure with biodiesel are used as containers and auto parts in direct contact with biodiesel fuel. Such materials are more affordable alternatives. In general, introducing polymers in biodiesel industry is intended to reduce the costs of raw materials and catalysts, enhance fuel characteristics, and avoid the negative impact in materials exposed with biodiesel. Such approach promotes sustainability and economic feasibility.

## 7. Future Outlook

Despite the current trend of biodiesel production from vegetative and animal feedstocks, promoting other biologically synthesized biodiesel from polymeric feedstocks would be an alternative track toward sustainable energy resources. Undoubtedly, production techniques need intensive development for a fast and better yield. Moreover, recent studies are highly recommended to enhance the drawbacks of poor physicochemical properties for polymeric biodiesels and reveal the toxicity of byproduct wastes. Although the polymeric catalysis is well investigated, it needs inducement toward naturally and waste derived catalysts since they have the advantage of being ecofriendly, less toxicity, and with lower costs. A substantial enhancement in biodiesel cold-flow properties was achieved by implanting polymeric CFIs. However, promoting the natural and low-costs polymeric materials are encouraged. Moreover, the effect of polymeric CFIs on the physicochemical properties other than the cold-flow properties lacks scientific investigations. Lastly, there is a necessity to expand research on the stability of polymeric materials exposed to biodiesel since it would help choosing appropriate polymers for auto parts and storage products, particularly those in direct contact with biodiesel fuel.

## 8. Conclusions

Polymers are used in a wide spectrum of our daily life. Alternative energy industry is one of the flourishing sectors that distinguishes the current and coming era of human history. In this context, polymers were introduced in bioenergy industry. The current review focused on the valorization of polymers in the biodiesel industry. Polymers were utilized in biodiesel production based on four approaches, i.e., polymeric biodiesel, polymeric catalysts, cold-flow improvers (CFIs), and stabilized exposure materials. Polymeric biodiesel is produced biologically from polymeric materials known as polyhydroxyalkanoates (PHAs), e.g., medium chain length PHAs (mcl PHAs) and poly-(R)-3-hydroxybutyrate (P3HB), which are sourced from materials rich in carbon. Those polymers are usually esterified to synthesize hydroxyalkanoates methyl ester (HAME) and hydroxybutyrate methyl ester (HBME) which are the polymeric biodiesel. Despite the similarity with vegetative and animal-based biodiesels, more studies are needed to develop the production techniques since the current method consumes much time and produces little yield with low cetane number. Polymers offer efficient acidic, basic, homogeneous, and heterogeneous catalysts for free fatty acids (FFA) esterification and triglycerides transesterification. In this review, polymeric catalysts were classified into three groups chemically synthesized, naturally derived, and waste derived polymeric catalysts. The last two groups are privileged with ecofriendly, reusable, non-toxic, and low-costs traits. Besides catalysis, polymers were utilized as CFIs for both pure biodiesel, such as polyolefins, ethylene/vinyl acetate copolymers, polyacrylates and related copolymers, and maleic anhydride copolymers and their derivatives, and blended biodiesel/diesel, such as Polyolefin (copolymers of C9–C22 α-olefins), EVA copolymer, poly(methyl acrylate), and poly(acrylic acid) and poly(tetradecyl methacrylate). The activity of polymers as CFIs comes from their capability to modify biodiesel agglomeration where the formed crystals flow easily due to the needle-like shape. Lastly, polymers are used in products directly in contact with biodiesel fuel, e.g., storage tanks and auto parts. Static immersion test is the tool used to verify the stability of polymeric materials toward biodiesel exposure. Teflon, Viton A401-C, Viton GFLT, nitrile rubber, polychloroprene, fluoro-Viton A, and polyoxymethylene (POM) are examples of polymers that showed insignificant change toward biodiesel exposure. However, high-density polyethylene (HDPE), polyamide 66 (PA66), polyethylene (PE), polytetrafluoroethylene (PTFE), ethylenepropylenediene monomer (EPDM), nylon 6/6, nitrile rubber, and high-density polypropylene showed instability once they were in direct contact with different sources of biodiesel fuel. Indeed, expanding the utilization of polymers toward biodiesel industry is promoted in accordance with the revolution of green chemistry.

## Figures and Tables

**Table 1:** Some examples of PHAs.

Type of Polymer	Type of PHAs	Source of Carbon	Synthesis Microbe	References
**Homopolymer**	P3HB	Waste glycerol	*C. necator* DSM 545	[37]
		Soy cake and molasses		[38]
		Sugar of coconut, palm, rock, and toddy	*C. necator* strain A-04	[39]
		Soybean oil	*R. eutropha* H16	[40]
		Pineapple crude glycerol	*Bacillus firmus* NII 0830	[41]
		Cooking oil	*Burkholderia thailandensis*	[42]
	PHHp	Heptanoate	*P. putida* KTOY06	[43]
	PHV	Undecanoic acid	*hydrophila* 4AK4	[44]
	PHDD	Sodium dodecanoate	*P. putida* KT2440	[45]
	PHO	Glycerol and sodium octanoate	*P. putida* ATCC47054	[46]
**Copolymer**	P3HB-*co*-HA	Gluconate alkanoates	*Pseudomonas* sp. 61-3	[47]
	P3HB-*co*-P4HB	n-alkanoic acids	*R. eutropha* H16	[48]
	P3HB-*co*-HHx	Lauric acid, and oleic acid	*A. hydrophila*	[49]
	P3HB-*co*-P3HV	Lactose, glucose and galactose	*P. hydrogenovora*	[50]
	P3HB-*co*-P3HV-*co*-P3HHx	Dodecanoic acid and propionic acid	Recombinant *A. hydrophila* 4AK4	[51]
	P(3HP-*co*-4HB	Glycerol	Recombinant *E. coli*	[52]

**Table 2:** Optimum values for polymeric biodiesel production HAME and MBME.

Polymeric Biodiesel	Polymers Source	Reaction Parameters					Yield (%)	References
		Alcohol	Catalyst	Ratio % (C in A) *	Temperature (°C)	Time (h)		
HAME	mcl PHA	Methanol	H_2_SO_4_	15	100	60	65	[35]
HAME	mcl PHA	Methanol	H_2_SO_4_	10	67	60	68	[20]
HBME	P3HB	Methanol	H_2_SO_4_	15	100	60	52	[35]
HBME	P3HB	Methanol	H_2_SO_4_	10	67	60	40	[57]
HBME	P3HB	Methanol	H_2_SO_4_	10	67	50	70.7	[21]
HBME	P3HB	Methanol	H_2_SO_4_	10	67	60	65	[22]

* C in A: catalyst in alcohol.

**Table 3:** The physicochemical properties of polymeric biodiesel (HBME) (adapted from [20,22,57]).

Physicochemical Properties	Unit	Value
Density at 20 °C	Kg/m^3^	900
Viscosity 20 °C	mm^2^/s	4
Pour point	°C	1
Flash point	°C	68.5
Heating value	MJ/kg	25.1
Cetane number	-	<1
Octane number (RON)	-	62.2
Oxygen content	%wt	41
Oxidative stability at 100 °C	h	8.13

**Table 4:** Some of the chemically synthesized polymeric catalysts.

Polymeric Catalyst	Biodiesel Feedstock	Yield (%)	Reusability		Reference
			Number of Cycles	Yield (%)	
FDU-15-SO_3_H	Soybean oil	99.0	4	90	[63]
PDVB-IL	Waste oils	99.0	6	89	[64]
Resin-N_3_=P(MeNCH_2_-CH_2_)_3_N	Soybean oil	100	5	90	[65]
Poly (butanesulfonic acid pyrrole) coated magnetic iron oxide	Waste oils	98.1	10	97.5	[66]
MIL-100(Fe)@DAILs	Oleic acid	93.5	6	86	[67]
HPW@MIL-100(Fe)	Acetic acid	96.3	5	95.5	[68]
Fe_3_O_4_@HKUST-1-ABILs	Soybean oil	92.3	5	85	[69]
HZnPS-1	Oleic acid	90	5	80	[70]
Oligocat	Swine tallow	96.5	3	96.5	[9,10]
Pd/HPS *	stearic acid	97	-	-	[71]

* This polymer-metal catalyst was used to catalyze hydrodeoxygenation reaction in purpose of synthesizing hydrocarbon-based biodiesel n-heptadecane.

**Table 5:** Some of the naturally derived polymeric catalysts.

Polymeric Catalyst	Biodiesel Feedstock	Yield (%)	Reusability		Reference
			Number of Cycles	Yield (%)	
CCu	Babassu oil	76.8	-	-	[73]
Chitosan immobilized with CaO	Soybean oil	97	-	-	[74]
Chitosan with sulfonic acid groups	Palmitic acid	89	4	80	[75]
HTCC/Na_2_SiO_3_/ECH	Soybean oil	97	7	83	[76]
Ferric alginate	Lauric acid	99	-	-	[78]
Tin (Sn^2+^) alginate	Oleic acid	98.7	8	97.6	[79]
CB-(AST-HPW)	Yellow horn oil	96	7	70	[80]
MSNC	Oleic acid	96	5	95	[25]
Lipase PS enzyme encapsulated with biopolymer κ-carrageenan	palm oil	100	5	82	[81]
ANL/UiO-66-PDMS-6 h	Soybean oil	88	10	83	[82]

**Table 6:** Some of the waste derived polymeric catalysts.

Polymeric Catalyst	Biodiesel Feedstock	Yield (%)	Reusability		Reference
			Number of Cycles	Yield (%)	
D50w2	Palmitic acid	76.8	7	59.8	[83]
D5081	Oleic acid	97	4	92	[84]
PSS	Oleic acid	53.4	3	35.1	[85]
sPS-S	Dodecanoic acid	100	10	88	[86]
MSA-Pani	Ricinoleic acid	92	2	89	[87]
p-TSA–PANI	Waste cooking oil	97.1	10	94.3	[88]
TPC-SO_3_H	Chicken fat	98.8	7	48	[23]

**Table 7:** Some polymeric CFIs for pure biodiesel.

Polymeric CFIs		Biodiesel	Concentration (wt%)	The Effect		Reference
				Property	Reduced Value (°C)	
Polyolefins	T803	Waste cooking oil	0.04	CP	1	[102]
	T803		0.04	CFPP	1	
	P388	Waste cooking oil	0.1	CFPP	2	[27]
	A134		0.1	CFPP	2	
	T803		0.1	CFPP	2	
	IX-248		0.1	CFPP	3	
	LZ-7749		0.1	CFPP	2	
	CS-1		0.1	CFPP	2	
	V-385		0.1	CFPP	2	
Ethylene/vinyl acetate copolymers	EVA	Waste cooking oil	0.02–0.08	CFPP	2	[102]
				PP	6	
		Soybean	0.01	PP	2	[104]
		Waste cooking oil	0.02–0.08	PP	3	[103]
		Canola	1	PP	11	[105]
	Keroflux (BASF), ethylene/vinyl acetate/acrylate	Blended rapeseed and soybean oil	0.3	CP	2	[28]
				CFPP	9	
Polyacrylates and related copolymers	Poly(dodecyl methacrylate)	Canola	1	PP	−46 *	[105]
	Poly(hexadecyl methacrylate)	Canola	1	PP	−20 *	
	polymethyl acrylate	Waste cooking oil	0.02–0.08	PP	8	[102]
Maleic anhydride copolymers and their derivatives	Poly(MA-alt-1-octadecene)	Palm oil	2	PP	6	[106]
	poly(MA-alt-1-octadecene)	Tobacco seed oil	1	CFPP	7	[107]
	octadecyl vinyl ether	Canola	1	PP	3	[105]

* These are the total values of PP.

**Table 8:** Some polymeric CFIs for blended biodiesel/diesel.

Polymeric CFIs	Biodiesel/Diesel Blend	Concentration (wt%)	Effect		Reference
			Property	Reduced Value (°C)	
Polyolefin (copolymers of C9–C22 α-olefins)	Waste cooking oil/diesel	0.04	CP	8	[30]
			PP	7	
EVA copolymer	Waste cooking oil/diesel (B20)	0.08	CP	8	[103]
			CFPP	11	
			PP	10	
	Rapeseed oil/diesel (B50)	320 ppm	CFPP	17	[108]
Poly(methyl acrylate)	Coconut/diesel (B20)	0.03	PP	9	[109]
			CP	3	
			CFPP	8	
Poly(acrylic acid) and poly(tetradecyl methacrylate)	Palm oil/diesel (B20)	0.1	PP	7	[29]

**Table 9:** Instable polymers toward biodiesel exposure.

Polymers	Biodiesel	Temperature (°C)	Time (Day)	Effect	Reference
HDPE	Soybean and sunflower biodiesel	60	125	Increase 5% weight	[113]
HDPE	Palm biodiesel and acidic-biodiesel blends.	55	98	Increase 5% weight	[17]
PA66	Palm biodiesel and acidic-biodiesel blends.	55	98	Decrease 2% weight	[17]
PE and POM	Biodiesel of palm, canola, soybean, and cotton	Room temperature	28	Decrease 1% weight	[114]
PTFE	Palm biodiesel and diesel	25	41.67	Decrease weight and volume	[32]
EPDM	Palm biodiesel and diesel	25	41.67	Weakness in hardness and tensile stress.	[32]
Nylon 6/6. Nitrile rubber. High-density polypropylene.	Biodiesel blends	51.7	28.92	Recorded effect in the physical properties.	[115]

**Table 10:** Stable polymers toward biodiesel exposure.

Polymers	Biodiesel	Temperature (°C)	Time (Day)	Effect	Reference
Teflon, Viton A401-C. Viton GFLT	Biodiesel blends	51.7	29	Insignificant change	[115]
Nitrile rubber. Polychloroprene, and fluoro-Viton A	Palm biodiesel	50	20.83	Insignificant change	[31]
POM	Palm biodiesel and acidic-biodiesel blends	55	98	Insignificant change	[17]

## Data Availability

Not applicable.
